# Growing your own in times of crisis: the role of home food growing in perceived food insecurity and well-being during the early COVID-19 lockdown

**DOI:** 10.35241/emeraldopenres.14186.2

**Published:** 2021-10-13

**Authors:** Bethan R. Mead, Jessica A. C. Davies, Natalia Falagán, Sofia Kourmpetli, Lingxuan Liu, Charlotte A. Hardman

**Affiliations:** 1 Department of Psychology, University of Liverpool, Liverpool, United Kingdom; 2 Lancaster Environment Centre, Lancaster University, Lancaster, United Kingdom; 3 Plant Science Laboratory, Cranfield University, Cranfield, United Kingdom

**Keywords:** urban agriculture, home food growing, well-being, food security, food insecurity, COVID-19, lockdown

## Abstract

Household food insecurity and poor well-being have increased during the coronavirus disease 2019 (COVID-19) pandemic and resulting lockdown measures. Home food growing has been associated with improved food access and well-being, but it is unknown what role it plays during food supply crises and lockdown. It is also unclear how home food growing and social restrictions may affect opinions about growing food in urban areas (i.e., urban agriculture [UA]).

A cross-sectional online survey was conducted during the UK national lockdown in March-April 2020 to measure home food growing, perceived food insecurity, well-being, and opinions of UA.  The participants were 477 UK-based adults (369 female, mean age 39.57 years ± 13.36); 152 participants were engaged in home food growing prior to the pandemic. Responses were compared to data collected from a separate sample of participants before the pandemic ( *N *= 583) to explore potential shifts in opinions about UA.

Participants who engaged in home food growing had lower levels of food insecurity ( *U *_­_= 19894.50, *z *= -3.649, *p*<.001, *r *= -.167) and higher well-being ( *U* = 19566.50, *z *= -3.666, *p*<.001, *r* = -.168) than those not engaged in home food growing. Perceived food insecurity partially mediated the relationship between home food growing and well-being; home food growing was associated with less food insecurity, which in turn was associated with better well-being. There were no differences in opinions of UA compared to the sample of participants from before the pandemic.

Home food growing may have had a protective effect over perceived food security and well-being in the early stages the pandemic. Opinions of UA were positive and unchanged compared to data collected pre-pandemic. Policies that support home food growing and access to suitable growing spaces and resources may be beneficial for food system resilience and well-being.

## Introduction

The global coronavirus disease 2019 (COVID-19) pandemic triggered widespread lockdown restrictions to limit the movement of citizens and control the spread of the virus ( Koo *et al*., 2020). These are predicted to have negative and long-lasting consequences for mental health ( Daly *et al*., 2020; Marroquín *et al*., 2020; Pierce *et al*., 2020; Rajkumar, 2020; Sani *et al*., 2020; Torales *et al*., 2020; Zhang & Ma, 2020). Evidence from the early stages of the pandemic indicates that the incidence of mental ill health and poor well-being have increased ( Daly *et al*., 2020; Pierce *et al*., 2020). Declines in well-being are likely to be compounded by pandemic-related isolation, fear, and existing health vulnerabilities experienced by some individuals ( Torales *et al*., 2020). Estimates suggest that up to 20% of the population in the United Kingdom (UK) will require mental health support as a result of this pandemic ( O’Shea, 2020). Therefore, as the pandemic continues, there is an urgent need to improve understanding of its impact on mental health and related issues.

Lockdown restrictions to slow the spread of the virus in the UK began in late March 2020. These included the closure of schools, non-essential retail and many businesses, limitations to citizens’ movements, shielding of vulnerable individuals and requirements for social distancing ( Brown, 2020). During the same time period, media reports highlighted disruptions to food supplies and food availability in supermarkets and shops, with purchasing restrictions placed on some items. Shoppers stockpiling food created surges in demand ( O’Connell *et al*., 2020). This rendered many foods unavailable, increasing reliance on highly-processed, often less-healthy food options ( Tan *et al*., 2020). Demand surges highlighted vulnerabilities in the UK food supply chain due to an over-reliance on a small number of EU countries for food imports and a reduced supplier base for supermarkets ( Garnett *et al*., 2020). Emerging evidence suggests that levels of food insecurity quadrupled during the same time period ( Loopstra, 2020). Food insecurity refers to limited access to safe, appropriate and nutritious food ( Taylor & Loopstra, 2016), and is robustly associated with poor mental health ( Frongillo *et al*., 2019; Jones, 2017; Pourmotabbed *et al*., 2020). In addition to food insecurity resulting from a lack of buying power, the lack of food in shops and lockdown restrictions on movement, which prevent people from obtaining food, appear to be driving new dimensions of food security ( Loopstra, 2020). Lack of food in shops, in particular, may explain up to 40% of food insecurity experiences during this time ( Loopstra, 2020). Thus, as intermittent tightening and relaxing of lockdown restrictions continues, there is an urgent need to understand potential factors that may mitigate the consequences of the pandemic on food insecurity and mental health.

Urban agriculture (UA), the growing of fruits and vegetables in urban and suburban areas, has historically been relied upon to bolster food supplies during times of crisis, such as war and food system shocks ( Mok *et al*., 2014). For example, growing food at home in “Victory Gardens” was encouraged to help buffer against food shortages during the Second World War ( Mok *et al*., 2014). Therefore, home food growing in gardens and allotments has been highlighted as a potential means of providing access to nutritious, healthy food in urban areas during the COVID-19 pandemic ( Lal, 2020). Existing literature from before the pandemic indicated that home food growing can supplement household food supplies and reduce food insecurity ( Algert *et al*., 2016; Galhena *et al*., 2013; Kortright & Wakefield, 2011). In addition to this, engagement in urban food growing (in general) has been associated with mental health benefits ( Audate *et al*., 2019; Kingsley *et al*., 2009; Lovell *et al*., 2014). Home food growing in particular has been associated with improved well-being ( Dobson *et al*., 2020; Genter *et al*., 2015; Soga *et al*., 2017a) and reduced stress ( Palar *et al*., 2019; Van Den Berg & Custers, 2011).

**Figure 1.**
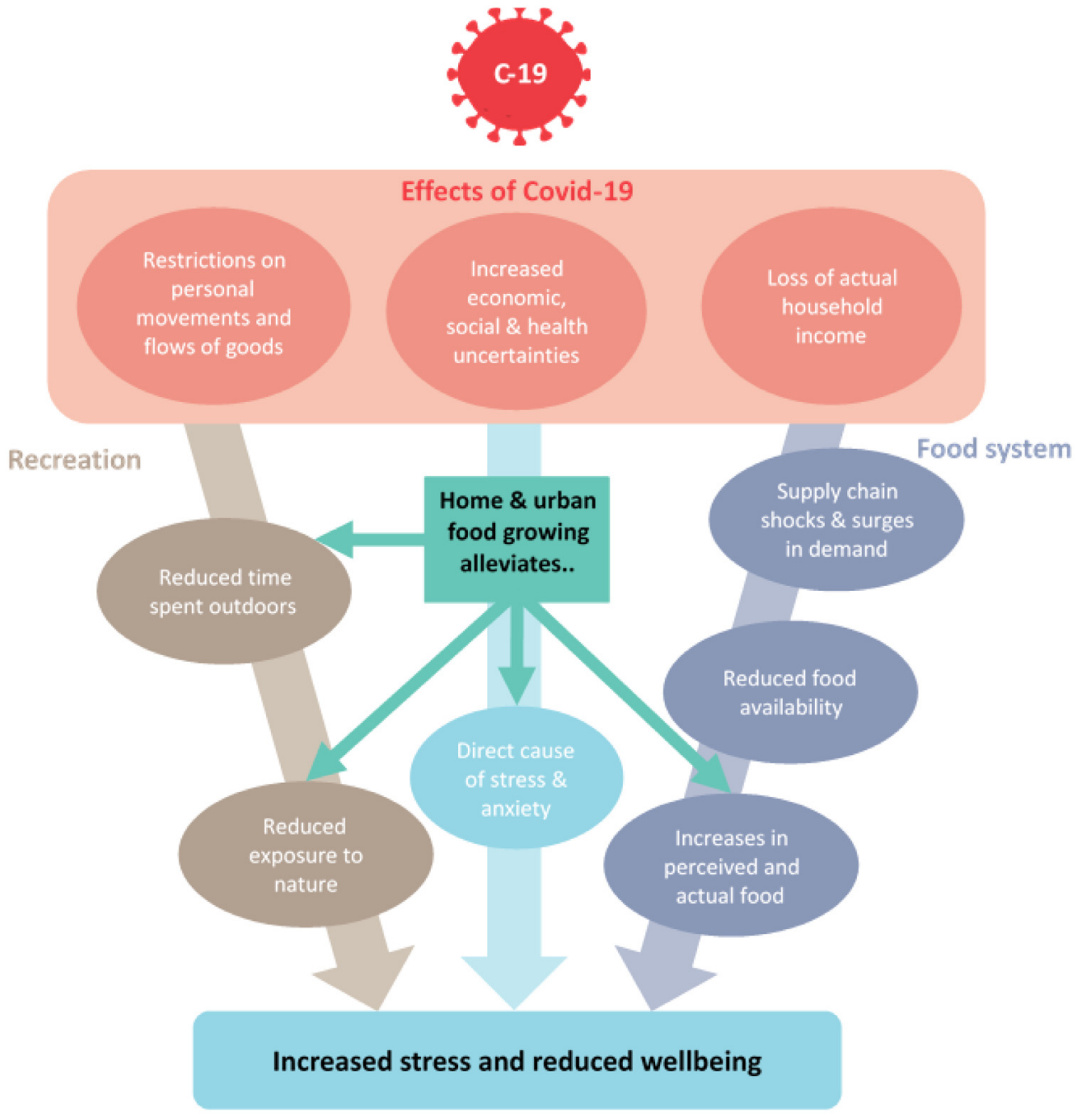
Conceptual illustration of how the pandemic may drive food insecurity and increased stress/reduced well-being, and where home food growing may impact this.

Taken together, existing evidence implies that home food growing could be beneficial in mitigating some of these negative impacts of the COVID-19 pandemic. However, to our knowledge, this has not been previously investigated in academic research. This issue is timely, as reports by the UK media and other global outlets during the pandemic indicate a surge in interest in home food growing. This has included reported seed shortages in garden centres, a rise in people taking up home food growing, and large increases in applications for allotment gardens. Home food growing could therefore represent a healthy, sustainable and accessible strategy for coping with the food insecurity and well-being impacts of the COVID-19 pandemic. Thus, the primary aim of the current study was to explore relationships between home food growing, food insecurity and well-being during the early stages of the COVID-19 pandemic. The proposed relationships are illustrated in Figure 1.

Whilst UA has been proposed as a viable means of reducing food insecurity ( Edmondson *et al*., 2019), less is known about how it is viewed by the general public. Public opinion is an important consideration in promoting the uptake of urban-grown food, particularly in household settings. Some studies have highlighted concerns about “unnaturalness” of food produced via UA ( Specht *et al*., 2016a), and contamination from urban pollutants ( Kim *et al*., 2014; Lal, 2020). However, increased interest in home food growing in the UK, albeit anecdotal, may also be accompanied by a shift in opinions of UA. A small, recent study of public opinions of urban farming in North America reported more positive than negative opinions ( Grebitus *et al*., 2020). A secondary aim of the current study was therefore to explore changes in opinions of UA by comparing data collected at the start of the UK lockdown with data on opinions of UA from a separate sample of participants in a study conducted before the pandemic (the pre-pandemic study; Mead *et al*., 2021b).

We predicted that people engaged in home food growing would report less perceived food insecurity (Hypothesis 1) and better well-being (Hypothesis 2) than those not engaged in home food growing. We also predicted that individuals with higher levels of perceived food insecurity would be more likely to report intentions to engage in home food growing (Hypothesis 3). Finally, we tested a non-directional hypothesis (Hypothesis 4) that participants in the current study would show differences in opinions of UA compared to participants in the pre-pandemic sample ( Mead *et al*., 2021b).

## Methods

### Participants

Participants were recruited via online adverts shared on social media and in online community groups and newsletters. We targeted social media/online groups and newsletters with either a food growing or a general focus (for example, general interest, UK-specific, or “help needed” sections of social media sites, groups and message boards). Recruitment was targeted in this way to ensure we recruited a mixture of participants who were and were not engaged in home food growing. Study adverts described the study as being about “food, well-being and the coronavirus outbreak”. Individuals who were eligible to participate were aged 18 years or older and based in the UK. A sample size calculation based on data from our pre-pandemic study ( Mead *et al*., 2021b) indicated that a sample size of 428 was required to detect small-medium effect with 95% power. Given the unprecedented nature of the pandemic, and the lack of previous research to inform a sample size calculation, we based this on a nondirectional analysis for detecting a difference in opinions of UA between the current study and the pre-pandemic study. Output of the sample size calculation is shown in the Extended data ( Mead, 2021).

### Measures

***Demographics.*** Participants reported their age (years), gender, which UK country they were currently residing in, and their height and weight. They indicated their ethnicity (Asian/Asian British; Black/African/Caribbean/Black British; Mixed/Multiple ethnic groups; Other; White; Prefer not to answer) and employment status (Employed full-time/Employed part-time/ Unemployed looking for work/ Unemployed not looking for work/ Retired/Student/Unable to work/ Homemaker/Voluntary employment/Prefer not to answer).

***Well-being.*** The World Health Organisation (WHO)-5 index was used to measure well-being ( Topp *et al*., 2015). Participants responded to five items that assessed subjective well-being over the previous 2 weeks (e.g., “I have felt cheerful and in good spirits”). Responses were scored 0 (“At no time”) to 5 (“All of the time”), summed and multiplied by four to give a total score out of 100. Higher scores indicate better well-being. Cronbach α value for the measure was .850. An attention check (please select “All of the time”) was inserted into this questionnaire to monitor for random responding.

***Experience of food insecurity.*** A modified version of the eight-item Food Insecurity Experience Scale (FIES; Smith *et al*., 2017) was used to assess perceived experiences of food insecurity. Items were adapted to assess the prevalence of aspects of food insecurity over the previous 7 days due to lack of food available in shops (e.g., “During the last 7 days was there a time you were worried you would run out of food because of a lack of food in the shops?”) as opposed to the typically assessed time period of the previous 12 months, and experiences related to lack of money ( Smith *et al*., 2017). The modified scale is shown in the Extended data ( Mead, 2021). This modification was designed to capture experiences specific to the early lockdown phase, when media reports suggested food shortages at UK shops and supermarkets. Responses were “Yes” or “No”. Scores were determined by summing the number of “Yes” responses. Higher scores indicated greater perceived experience of food insecurity. Cronbach α value for the modified scale was .748.

***Home food growing.*** We used the following question to determine if participants were engaged in home food growing: “Is growing your own fruits and vegetables something you…”. Participants selected one of the following response options: i) already do, ii) don’t currently do but are planning to do, iii) are not planning to do but would consider in the future, and iv) are not planning to and would not consider doing. Participants who responded “i) already do” were categorised as engaged in home food growing (Growers); other responses were categorised as Non-growers.

***COVID-19 related questions.*** Participants provided the following information in relation to the UK lockdown: keyworker status ^1^ (yes/no), isolation status (social distancing/self-isolating/ lockdown/working out of home but social distancing/life as normal/other). They also indicated if they were in a high-risk category for coronavirus, and if they or someone in their household had experienced symptoms of, or been diagnosed with, coronavirus.

Additional questions regarding participants’ concerns about accessing food were asked to provide more detail about the types of foods that they were concerned about accessing, if any. Participants reported if they had concerns about accessing food (yes/no). Next, they indicated if they had concerns about accessing specific types of food (fruit and vegetables/meat/dairy products/dried foods/bread/baby or infant food/pet food/other). Finally, participants were asked to indicate which extra measures they may be taking to ensure adequate food supply, if any (buying extra food/sharing food with others/monitoring or reducing the food I buy/monitoring or reducing the food I eat/growing my own fruits and vegetables/eating fruits and vegetables somebody else grows/sourcing food elsewhere/other).

***UA opinion questions.*** Participants read the following definition of UA: “Urban agriculture means growing fruit and vegetables in urban, suburban and surrounding areas.” This was followed by the question “Do you think urban agriculture could be beneficial/not beneficial to any of the following?” and the following items: i) You, your family and friends; ii) Your community; iii) The environment; iv) The economy; v) Society in general; vi) Entertainment/alleviate boredom; vii) For education/home-schooling; viii) Alternative income because of unemployment; ix) Ensuring my family and I can eat healthily; x) Ensuring social distancing by accessing food without being in contact with others. Response options were beneficial/not beneficial/unsure. Items i-v were taken from ( Mead *et al*., 2021b); items vi-x were added to assess opinions of UA related to the pandemic.

### Procedure and design

The survey was hosted via Qualtrics (Provo, Utah) and accessed via a weblink placed in study advertisements. Participants read an information sheet (Participant Information Sheet), provided informed consent, then indicated their location (country). The survey was terminated if any country other than the UK was selected. Demographic questions, FIES, WHO-5 and COVID-related questions were then displayed in a random order with UA opinion questions always at the end. Links to the UK government website for definitions of key worker, social distancing, self-isolation and lockdown were provided next to the related questions. “Lockdown” was added as a category for isolation status alongside social distancing and self-isolation due to a change in UK law and terminology used by the UK Government shortly before the survey launched ( Brown, 2020). Finally, participants were asked if they had taken part in the pre-pandemic study. This question was asked as we planned to compare data on opinions of UA between the current study and our previous study, and we aimed to avoid any overlap in participants.

A debrief sheet was displayed at the end of the study, and participants were invited to enter a prize draw to win a £20 shopping voucher as thanks for their time. The survey took approximately ten minutes to complete. The study was approved by the University of Liverpool Research Ethics Committee, ref: 5383. All data were collected between 25/03/20 and 07/04/20.

### Analysis

Data were analysed using SPSS 24 (IBM Corp., Armonk, New York, USA). Scale scores were calculated in accordance with author instructions and as described above. Significance was set at *p*<.05 for statistical tests, except for those where it was adjusted to *p*<.01 or *p*<.005 to correct for multiple comparisons. Exploratory mediation analysis was conducted in PROCESS version 3.5 for SPSS ( Hayes, 2018) with bias corrected bootstrapping (1000 samples) for analysis of indirect effects, with 95% confidence intervals reported.

For analyses that compared participants engaged in home food growing to those who were not engaged in home food growing, we defined Growers and Non-growers as described above (see *Home food growing*). Scores on the FIES and WHO-5 violated the assumption of normality so nonparametric analysis was used to test for differences in food insecurity and well-being between Growers and Non-growers (Hypothesis 1 and 2). A multinomial logistic regression was used to test if participants with greater experience of food insecurity would be more likely to report intentions of engaging in home food growing (Hypothesis 3).

Non-directional analyses were conducted to test if opinions of UA had changed from before the COVID-19 pandemic to the early COVID-19 lockdown (Hypothesis 4). Responses to the UA opinion questions from the current study were compared to responses to the same questions from the pre-pandemic study. Responses from the pre-pandemic study were collected in summer 2019, before the COVID-19 pandemic began. Chi-Square tests were used to test for differences between responses to UA opinion questions and studies (current study; Mead *et al.*, 2021b). The significance threshold for these analyses was adjusted to *p*<.01 to correct for multiple comparisons. An additional exploratory analysis was conducted to assess if responses to UA opinion questions were associated with participants being classed as a Grower or Non-grower ( *p*<.005 to correct for multiple comparisons). Significant Chi-Square tests were explored further by examination of standardised residuals.

## Results

### Sample characteristics

In total, 684 participants entered the survey. Of these, 523 participants reached the end of the survey; 161 were excluded due to their only partial completion of the study or their location being outside of the UK. Responses with missing data or where participants failed the attention check were also excluded, leaving a final sample of 477 participants.

Most participants were female (369, 77.4% female), White (92%) and employed full time (56.8%) ( Table 1). Participants had an average age of 39.57 years ( *s.d.*= 13.36, *range* 18–82). Mean body mass index (BMI) was 26.41kg/m ^2^ ( *s.d.* = 6.15, range 16.55–57.45). One-hundred and fifty-two participants (31.9%) were already engaged in home food growing (see Table 1). Additional demographic information for the sample is shown in Table 1. Across the different categories of grower status, participants were well matched for demographic characteristics (gender, employment status, ethnicity, location within UK – Table 1), with the exception of age; participants who reported their grower status as i) already engaged in home food growing were significantly older than those who reported their status as iii) not planning grow their own fruits and vegetables to but would consider doing so (42.82 years ± 13.73 versus 37.17 years ± 13.13, respectively, *p* = .001). Comparison for age between other categories of grower status, and for other demographic variables, were not significant ( *p*>.05). These analyses and a breakdown of demographic information by grower status are shown in the Extended data and Tables S1 and S2 ( Mead, 2021).

**Table 1.** Participant characteristics, coronavirus isolation status and grower status.

Characteristic		*n*	*%*
Gender	Female	369	77.4
	Male	105	22.0
	Other	3	0.6
Location	England	379	79.5
	Scotland	28	5.9
	Wales	68	14.3
	Northern Ireland	2	0.4
Ethnicity	Asian/Asian British	12	2.5
	Black/African/Caribbean/Black British	2	0.4
	Mixed/Multiple Ethnic Groups	12	2.5
	Other	6	1.3
	White	439	92.0
	Prefer not to answer	6	1.3
Employment Status	Employed full-time	271	56.8
	Employed part-time	74	15.5
	Unemployed and looking for work	7	1.5
	Unemployed and not looking for work	5	1.0
	Retired	25	5.2
	Student	52	10.9
	Unable to work	21	4.4
	Homemaker	13	2.7
	Voluntary employment	4	0.8
	Prefer not to answer	5	1.0
Isolation Status	Social distancing	49	10.3
	Self-isolating	44	9.2
	Lockdown	319	66.9
	Working out of home but social distancing	45	9.4
	Life as normal	1	0.2
	Other	19	4.0
Grower status	Already do (Grower)	152	31.9
	Don’t currently do but are planning to do (Non- grower)	63	13.2
	Are not planning to do but would consider in the future (Non-grower)	165	34.6
	Are not planning to do and would not consider doing (Non-grower)	97	20.3

Most participants reported engaging in some form of COVID-19-related isolation measures. Overall, 98 participants (20.5%) identified as keyworkers, 267 participants (56%) reported being concerned about their access to food, and 358 participants (75.1%) reported taking extra measures to ensure they had enough food during the pandemic. Additional sample descriptives regarding COVID-related questions are shown in the Extended data ( Mead, 2021; Tables S1–5).

### Home food growing, well-being and experience of perceived food insecurity

The mean score for well-being (WHO-5) for the full sample was 44.35 ( *s.d.*=20.41, *range* 0–100), indicating moderate levels of well-being. The mean score for perceived food insecurity (FIES) for all participants was 1.21 ( *s.d.*=1.63, *range* 0–8), indicating low levels of perceived food insecurity, on average. There was a significant small-moderate, negative correlation between well-being and perceived food insecurity, *r*=-.231, *p*<.001, *r ^2^*=.053. Greater levels of perceived food insecurity were associated with lower levels of well-being.

Growers had significantly lower scores for perceived food insecurity (FIES; *Mdn* = 0.00) than Non-growers ( *Mdn* = 1.00), *U* = 19894.50, *z* = -3.649, *p*<.001, *r* = -.167, thus supporting hypothesis 1. Scores for well-being (WHO-5) were significantly higher for Growers ( *Mdn* = 48.00) than Non-growers ( *Mdn*=44.00), *U* = 19566.50, *z* = -3.666, *p*<.001, *r* = -.168, thus supporting hypothesis 2.

### Mediation analysis

**Figure 2.**
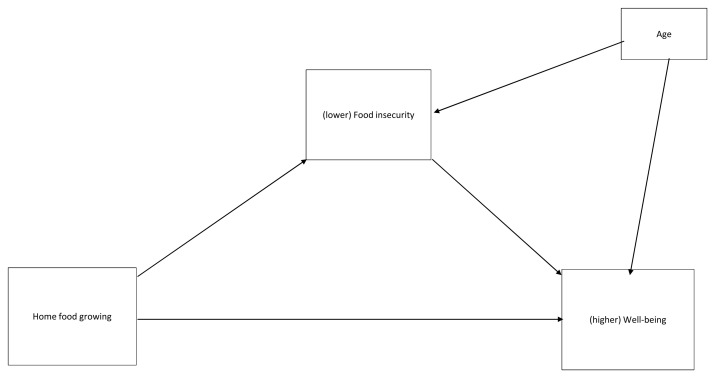
Proposed mediation model.

We conducted an exploratory, simple mediation analysis ( Figure 2) to test if perceived food insecurity (FIES score) explained the relationship between home food growing and well-being (WHO-5 score). Age (years) was entered as a covariate given the finding that participants in two categories of grower status differed in age. Associations between the variables are shown in Table 2. Overall *R ^2^* for the model was .110. Being engaged in home food growing (versus not engaged) was associated with lower food insecurity, and lower food insecurity was associated with better wellbeing. There was a significant indirect effect of home food growing on wellbeing via food insecurity ( *IE*=.914, *SE* = .473, *CI* .184 to 2.016). The direct relationship between home food growing and wellbeing was also significant, such that being engaged in home food growing was associated with better well-being. This indicates that, when controlling for age, the relationship between home food growing and well-being was partly mediated by food insecurity.

**Table 2.** Direct associations between variables

*Association*	*b (SE)*	*p*	*95% CI*
Home food growing → Food insecurity	-.425 (.158)	<.001	-.735 to -.116
Food insecurity → Well-being	-2.148 (.561)	<.001	-3.250 to -1.045
Home food growing → Well-being	5.346 (1.940)	.006	1.533 to 9.158
Age → Food insecurity	-. 023 (.006)	< .001	-.034 to -.012
Age → Well-being	.294 (.069)	<.001	.160 to .429

Note. CI =confidence intervals; SE = standard error

### Food insecurity and intention to engage in home food growing

Data from participants categorised as Non-growers ( *n* = 325) were used to test if those with greater experience of food insecurity would be more likely to report intentions of engaging in home food growing (Hypothesis 3). Data were split by Non-grower response type: i) did not currently grow their own fruits and vegetables but were planning to do; ii) were not planning to grow their own fruits and vegetables to but would consider doing so; or iii) were not planning to grow their own fruits and vegetables and would not consider doing so. Data were entered into a multinomial logistic regression with FIES score (food insecurity) as the predictor and grower status as the outcome, model χ ^2^ (2) = 1.297, *p*=.523. The category “iii) not planning to do and would not consider doing so” was the reference category as these participants reported having no intention of engaging in home food growing.

FIES score did not significantly predict membership of either category of intention to engage in home food growing ( *p*’s>.05). Therefore, experience of food insecurity did not predict immediate or future intentions to engage in home food growing. Hypothesis 3 is not supported. The regression table for this analysis is shown in Extended data, Table S6 ( Mead, 2021).

### UA opinion questions

To test for differences in opinions of UA we compared responses to UA opinion questions i) – v) from participants in the current study to responses to the same questions by UK-based participants in the pre-pandemic study ( Mead *et al*., 2021b). Data from participants who reported taking part ( *n*=14) or being unsure if they took part ( *n*=19) in the pre-pandemic study were excluded from this analysis. Data from the remaining 444 participants and the participants from the pre-pandemic study were analysed using Chi-Square tests to determine if there was an association between study (current study, pre-pandemic study) and proportion of participants indicating that they thought UA could be beneficial/not beneficial/unsure to the following: i) You, your family and friends, ii) Your community, iii) The environment, iv) The economy, and v) Society in general. Descriptive characteristics for participants from the pre-pandemic study and results of these analyses are shown in the Extended data and Table S7–8 ( Mead, 2021).

There were significant differences between study and responses to item iv) “the economy” ( *χ ^2^* (2) = 8.853, *p*=.002), and between study and responses to the item “society in general” ( *χ ^2^* (2) = 10.628, *p*=.005). We inspected standardised residuals to determine what may be driving these associations and found that these did not reach the adjusted threshold for statistical significance ( *z <* 2.58; *p‘s* > .01). These analyses are reported in the Extended data ( Mead, 2021). There was no evidence for differences between study and responses to the other items ( *p*’s>.01). These results therefore do not indicate any shift in opinion of UA between the pre-pandemic study and the current study. Overall, opinion of UA appeared to be positive across both studies: the proportions of participants endorsing UA as beneficial for items i)-v) ranged from 68.2–94.1% (Table S8).

### Exploratory analyses

Chi-Square tests were used to test whether membership of the categories Grower or Non-grower was associated with responses to UA opinion questions i)-x). In addition to items i)-v) listed above, participants indicated if they thought UA could be beneficial to vi) Entertainment/alleviate boredom, vii) For education/home-schooling, viii) Alternative income because of unemployment, ix) Ensuring my family and I can eat healthily, and x) Ensuring social distancing by accessing food without being in contact with others. An adjusted *p* value of *p*<.005 was used for these analyses to correct for multiple comparisons. There was a significant association between grower category and response to the item vi) Entertainment/alleviate boredom ( *χ ^2^* (2) = 12.575, *p* = .002). However, differences in proportions of participants selecting beneficial/not beneficial or unsure between the grower categories did not reach the adjusted threshold for statistical significance ( *p*>.005). No other associations between grower category and response to UA opinion questions were significant ( *p*’s>.005), therefore is no evidence that membership of the Grower or Non-grower category was associated with opinion of UA. Opinion of UA appears to have been high across Growers and Non-growers: the proportions of participants endorsing UA as beneficial for items i)-x) ranged from 50.5–96.7%. Results of these analyses are shown in the Extended data, Table S9 ( Mead, 2021).

## Discussion

The current study provides, to our knowledge, the first data on the relationships between home food growing, perceived food insecurity and well-being during the early stages of a national COVID-19 lockdown. Those engaged in home food growing reported less food insecurity and better well-being than those not engaged in home food growing. In an exploratory analysis, we showed that engaging in home food growing was associated with better well-being. This relationship was partially mediated by perceived food insecurity, such that engaging in home food growing was associated with less food insecurity, which in turn was associated with better well-being. The analysis also showed a direct association between food growing and wellbeing which was not explained by perceived food insecurity, indicating that there are other drivers of this relationship beyond those measured in the current study. Importantly, these effects were significant when age was controlled for. We also found that food insecurity did not predict immediate or future intentions to engage in home food growing. Finally, we saw no evidence for a difference in opinions of UA between the current study and data collected before the pandemic, or for differences in opinions of UA between participants who were or were not already engaged in home food growing.

Considering our findings, we tentatively suggest that home food growing may have had some form of protective effect over food insecurity and well-being during the early stages of the UK COVID-19 lockdown. Our findings of reduced levels of food insecurity and better well-being for Growers compared to Non-growers, combined with our finding that the relationship between home food growing and well-being was partially mediated by lower food insecurity, suggest that home food growing could be a protective factor in the face of challenges to food security and well-being posed by the pandemic (or other future crises). Previous research has shown that UA/home food growing has benefits for food insecurity and well-being ( Algert *et al*., 2016; Galhena *et al*., 2013; Genter *et al*., 2015; Kortright & Wakefield, 2011; Soga *et al*., 2017a). Others have theorised that these effects would be evident in the face of food system shocks ( Galhena *et al*., 2013). The current study extends these findings and suggestions. The UK was in the early stages of lockdown when these data were collected, and levels of food insecurity due to lack of access to food had dramatically increased across the UK ( Loopstra, 2020). Our observation that home food growing may provide a protective effect over well-being and food insecurity during this time provides novel evidence that home food growing may help to mitigate the negative consequences of food system shocks, such as those characterised by the disruption faced in the UK during March-April 2020. Food insecurity and poor well-being are continuing concerns, especially as the impact of Brexit and upcoming reductions to Universal Credit are predicted to exacerbate inequalities further and disproportionately impact on vulnerable groups ( Barons & Aspinall, 2020; Goodwin, 2021). The current study then provides an impetus for exploration of policy mechanisms that can support adoption of home food growing, with the aim of protecting and increasing resilience to food system shocks, and supporting access to fruits and vegetables.

Reductions in well-being have been reported during the early stages of the pandemic, and are predicted to persist as lockdown restrictions continue ( Daly *et al*., 2020; Pierce *et al*., 2020). Projections of the scale of the negative impact that the pandemic will have on well-being indicate large increases in the numbers of people within the UK requiring support for mental health issues ( O’Shea, 2020). This has prompted calls for innovative, efficacious interventions to support well-being in the context of the pandemic ( Holmes *et al*., 2020). Thus, findings from the current study suggest that home food growing should be investigated as a means of supporting well-being during the pandemic, although longitudinal data are needed to confirm this and identify the underlying mechanisms that might explain this effect. The restorative impact of time spent in nature and opportunity for physical activity associated with gardening activities, alongside or in addition to the act of food growing, may have also contributed to the increased well-being reported by Growers in this study ( Soga *et al*., 2017b). Nonetheless, our finding of greater self-reported well-being among Growers compared to Non-growers is consistent with existing research that has highlighted the potential benefits of UA and home food growing for well-being ( Audate *et al*., 2019; Howarth *et al*., 2020; Kingsley *et al*., 2009; Lovell *et al*., 2014). Efforts to facilitate the uptake of home food growing to maximise its potential benefits for well-being, such as national campaigns or subsidies to provide equipment, could benefit from capitalising on the increased public interest in home food growing that has been (anecdotally) reported by UK media outlets.

It should be noted that the March-April period when these data were collected was early in the UK growing season, so large food harvests from home food growing may have been unlikely. The protective effect of home food growing that we tentatively propose may be due to home food growing providing reassurance during an acute food system shock (early lockdown), rather than an immediate food source. Participants who reported already being engaged in home food growing may have been reassured by the expectation of what they would be able to harvest in the future should purchasing restrictions in supermarkets continue. It may also be that these individuals are better networked in local food growing communities, affording them better knowledge and access to less disturbed local food sources. However, we did not measure the scale of the growing that people were engaged in, or if they engaged in home food growing for subsistence or a hobby, and if this changed in response to the pandemic. Future work needs to consider this, as the amount of food that home food growing is expected to produce may moderate these effects.

We found no evidence that participants with higher levels of perceived food insecurity had immediate or future intentions of engaging in home food growing. This has implications for intervention development because home food growing has been shown to have benefits for tackling food insecurity ( Algert *et al*., 2016; Galhena *et al*., 2013; Kortright & Wakefield, 2011). Participants in this study may have been unaware of such potential benefits, or even felt discouraged from engaging in home food growing because of perceived barriers such as limited time, space, knowledge or financial resources ( Mead *et al*., 2021a; Schupp *et al*., 2016). Future home food growing interventions may wish to specifically target these individuals to address any barriers, both perceived and real, and raise awareness of the potential benefits of home food growing for tackling food insecurity, as these individuals may benefit from a home food growing intervention that is accessible and easily available.

These findings have implications for the wider understanding of the consequences of the COVID-19 pandemic. Food insecurity has been identified as an underlying factor in a “global food syndemic” that may exacerbate the impact of the COVID-19 pandemic ( Huizar *et al*., 2021). Huizar *et al*. highlight that food insecurity is linked to malnutrition and obesity, which in turn have been identified as risk factors for coronavirus infection and greater severity of the disease ( Gao *et al*., 2020). Thus, effective measures to reduce food insecurity may have an indirect effect on disease vulnerability via associated levels of malnutrition and obesity. Tackling food insecurity requires a multi-actor approach and is beyond the scope of this work, however the current study highlights the potential for home food growing to have benefits for perceived experience of food insecurity during the pandemic. In addition to this, home food growing and UA in general have been associated with better diet quality and reduced likelihood of having overweight or obesity during non-pandemic times ( Alaimo *et al*., 2008; Kamphuis *et al*., 2006; Mead *et al*., 2021b; Palar *et al*., 2019; Zick *et al*., 2013). With future lockdowns and social restrictions likely, home food growing should be investigated as a sustainable, holistic option for interventions that mitigate the negative consequences lockdown and the pandemic may have for food security and, indirectly, obesity, malnutrition and disease vulnerability.

Our secondary aim was to explore if opinions of UA may have shifted from 2019 ( Mead *et al*., 2021b) to the early stages of the pandemic. We also tested for differences in opinion of UA between Growers and Non-Growers. In the face of global (COVID-19), environmental, and political (Brexit) challenges to the UK food system, urban-grown food represents an innovative, sustainable solution to safeguarding the supply of healthy, nutritious food for the population ( Edmondson *et al*., 2019, Edmondson *et al*., 2020; Kourmpetli *et al*., 2020). Furthermore, the pandemic has highlighted shortcomings and inequalities in the UK food system ( Power *et al*., 2020). Understanding the impact on opinions of UA could help to inform future efforts to expand UA as a food systems solution. However, we saw no evidence that the COVID-19 pandemic or grower status were associated with opinions of urban grown food. Opinion of UA appeared to be generally positive both before and at the early stages of the pandemic, and regardless of participants’ engagement in home food growing during the early UK lockdown. The consistently positive opinion of UA in our UK-based studies is promising for efforts that aim to increase the uptake of urban-grown food. This suggests that neither the pandemic nor participants’ engagement in home food growing have had any negative effect on opinions of UA. However, specific concerns relating to urban-grown food, such as quality and safety, still need to be identified and addressed as part of efforts to upscale the UK’s urban-grown food production ( Kim *et al*., 2014; Kourmpetli *et al*., 2020; Specht *et al*., 2016b). 

### Limitations and future directions

Longitudinal data are needed to assess how home food growing affects well-being and perceived experience of food insecurity as the pandemic progresses. The cross-sectional nature of this study means we are unable to test the longevity of the effects reported here. Future work should consider assessing home food growing, well-being and food security at multiple time points to see if these effects persist as the pandemic continues. With future lockdowns and social restrictions likely ( Brown, 2020), innovative, sustainable activities will be needed to help mitigate the negative consequences of the pandemic. 

Although home food growing is considered a form of UA, it should be noted that we did not assess if participants resided in urban or rural areas. We are therefore unable to confirm if participants who reported engagement in home food growing were doing so in urban areas. Others have reported associations between home food growing and food security in rural settings ( Rammohan *et al*., 2019), therefore, our conclusions should be considered as limited to the impact of home food growing itself, rather than the wider practice of UA. Future studies should address this limitation by considering potential differences in the experiences of urban- and rural-based participants and if this might impact on their opinions of UA. 

We did not assess if Growers were engaged in home food growing prior to the pandemic, or if they had recently become engaged in home food growing response to lockdown (new Growers). It is possible that the lack of distinction may have obscured any subtle differences between the experiences or characteristics of such types of Growers. Furthermore, we did not assess the scale or longevity of participants’ engagement with home food growing and if this influenced our findings. Future work may wish to consider if large-scale, established home food growing, such as allotment growing, may have a different effect on well-being and food insecurity than small-scale, home garden-based growing.

We did not measure socioeconomic status (SES) of our participants, which is an additional limitation because SES could account for the associations between home food growing and lower food insecurity/better well-being. Importantly, participants in the different categories of growing status were well-matched for other demographic characteristics and did not differ in employment status. Due to a difference in age between two categories of grower status, age was controlled for in the mediation model and the relevant significant associations remained. Furthermore, controlling for age and SES in our previous study did not attenuate associations between urban agriculture, diet quality and potential mediators ( Mead *et al*, 2021b). Therefore, we tentatively suggest that differences in SES between participants are unlikely to fully explain our results though future work is needed to confirm this.

It should be noted that levels of perceived experience of food insecurity in our sample were low. Although we detected group differences in food insecurity between Growers and Non-growers, these findings may not be generalisable to participants or settings with high levels of food insecurity. We also adapted our measure of food insecurity (the FIES) to assess experience of food insecurity dues to a lack of food in shops over the preceding two weeks. Thus, we are unable to ascertain if the experiences of food insecurity reported by our participants were acute or chronic. The experience of food insecurity reported by participants in the current study may not be comparable to that of individuals with the highest levels of chronic food insecurity, therefore a more diverse sample is needed to extend these findings.

Finally, participants in the current study were predominantly white and female, thus limiting the generalisability of these findings to more diverse groups. Although mean BMI for this sample (26.36kg/m ^2^) was similar to the estimated average BMI for England (27.5kg/m ^2^), it is unclear if people who report being white or female are more or less likely than those who report other genders and ethnicities to engage in home food growing, and if this could have impacted results. Participant demographic data in the current study showed a similar distribution of gender and ethnicity to other studies in the literature that report similar effects (e.g. Kingsley *et al.*, 2009; Mead *et al.*, 2021b; Van Den Berg & Custers, 2011; although see Alaimo *et al.*, 2008), however, this research should be repeated with a more representative sample of participants to ascertain if the findings reported in the current study are applicable to other groups.

## Conclusion

Home food growing may have had a protective effect on levels of well-being and perceived experience of food insecurity at the start of the UK COVID-19 lockdown. Those engaged in home food growing reported higher levels of well-being and lower levels of food insecurity than those who were not engaged in home food growing. Home food growing was associated with lower food insecurity, which, in turn, was associated with better well-being. Opinions of UA were generally positive before and during the early stages of the pandemic, and regardless of participants’ engagement in home food growing. These results suggest that home food growing may have benefits for well-being and food security during lockdown, but longitudinal assessment of this as the pandemic progresses is needed to confirm this. Upscaling home food growing by increasing public interest and facilitating engagement in growing by making such opportunities more accessible could have tangible benefits for mitigating the impact of the COVID-19 pandemic on well-being and food security. Policies that provide access to land, skills, and opportunities for food growing should be developed to utilise the potential beneficial effects of home food growing for food system resilience, food security, and individual well-being.

## Data availability

### Underlying data

OSF: Home food growing, well-being and food security during the COVID-19 UK lockdown. https://doi.org/10.17605/OSF.IO/7EZJQ ( Mead, 2021)

This project contains the following underlying data:

- Home food growing lockdown study data Mead.sav
- pre-pandemic UA opinon comparison.sav

### Extended data

OSF: Home food growing, well-being and food security during the COVID-19 UK lockdown. https://doi.org/10.17605/OSF.IO/7EZJQ ( Mead, 2021)

This project contains the following extended data:

- Mead et al HFG, well-being, FIS - Extended data.docx (Supplementary analyses, additional sample information and copies of the questionnaires completed by participants)

Data are available under the terms of the Creative Commons Attribution 4.0 International license (CC-BY 4.0).
