# Supplementary material for: Growing your own in times of crisis: the role of home food growing in perceived food insecurity and well-being during the early COVID-19 lockdown
Source: Emerald Open Res. 2021 Oct 13;3:7. doi: 10.35241/emeraldopenres.14186.2 (PMC8547538; doi:10.35241/emeraldopenres.14186.2)
Supplement: Peer review history

# Peer review: Reviewer response for version 2

**DOI:** 10.21956/emeraldopenres.15504.r27782

**Published:** 2021-10-13

**Authors:** Pierre-Paul Audate

**Affiliations:** 1 Graduate School of Land Management and Regional Planning, Faculty of Planning, Architecture, Art and Design, Laval University, Quebec, QC, Canada

The authors addressed my concerns in the revised manuscript. I have no further comments to make.

Is the argument information presented in such a way that it can be understood by a non-academic audience?

Yes

Could any solutions being offered be effectively implemented in practice?

Yes

Is the work clearly and accurately presented and does it cite the current literature?

Yes

If applicable, is the statistical analysis and its interpretation appropriate?

I cannot comment. A qualified statistician is required.

Is real-world evidence provided to support any conclusions made?

Yes

Are all the source data underlying the results available to ensure full reproducibility?

Yes

Is the study design appropriate and is the work technically sound?

Yes

Are the conclusions drawn adequately supported by the results?

Yes

Does the piece present solutions to actual real world challenges?

Yes

Are sufficient details of methods and analysis provided to allow replication by others?

Yes

Reviewer Expertise:

Urban agriculture, Urban food systems, Urban greening, Urban planning, Urban heat island, sustainable development, Climate change

I confirm that I have read this submission and believe that I have an appropriate level of expertise to confirm that it is of an acceptable scientific standard.

# Peer review: Reviewer response for version 1

**DOI:** 10.21956/emeraldopenres.15274.r27671

**Published:** 2021-09-21

**Authors:** Aimee Pink

**Affiliations:** 1 Institute of High Performance Computing, Agency for Science, Technology and Research, Singapore, Singapore

Overall, this is a very well written manuscript with a particularly clear method and results section. The study examines the role home food growing had on well-being and food insecurity during the UK national lockdown due to the COVID-19 pandemic. Understanding the impact home grown could have is important for both the current pandemic, potential future pandemics but also general well-being and food insecurity.

**Methods**

 Participants – expansion on recruitment from the general online groups may be useful. For example, were they community groups from certain areas and therefore were a number of areas targeted? Did participants have to be from an urban or suburban area? Wondering if this may impact on their views of UA. May recruitment from these groups also be a limitation, leading to a predominantly white and female sample?

 Analysis – there is repetition from the measure section for home food growing definition, given it is a short manuscript and the definitions are clearly articulated, one definition may be sufficient.

**Results**

 Food insecurity and intention… - It may be worth removing “Non-grower” where you identify category iii as the reference group as here all categories are “non-growers”.

**Discussion**

 I am unsure if there are differences, but are males or females more likely to home-grow food? It is mentioned as a limitation but wondering how much this may influence findings.

 It has been mentioned several times but may be worth further reiterating that food insecurity continues to be of concern due to Brexit and changes to universal credits etc. It may be worth further emphasis the continued/broader applications of this study.

**General**

 It may just be the rendering/size of the image but the blue/white text in the middle of Figure 1 is difficult to read

Is the argument information presented in such a way that it can be understood by a non-academic audience?

Not applicable

Could any solutions being offered be effectively implemented in practice?

Yes

Is the work clearly and accurately presented and does it cite the current literature?

Yes

If applicable, is the statistical analysis and its interpretation appropriate?

Yes

Is real-world evidence provided to support any conclusions made?

Yes

Are all the source data underlying the results available to ensure full reproducibility?

Yes

Is the study design appropriate and is the work technically sound?

Yes

Are the conclusions drawn adequately supported by the results?

Yes

Does the piece present solutions to actual real world challenges?

Yes

Are sufficient details of methods and analysis provided to allow replication by others?

Yes

Reviewer Expertise:

Eating behaviours, food insecurity

I confirm that I have read this submission and believe that I have an appropriate level of expertise to confirm that it is of an acceptable scientific standard.

# Response

**Published:** 2021-10-05

**Authors:** Bethan Mead

**Affiliations:** University of Liverpool, United Kingdom

Thank you for these helpful comments and suggestions. We have uploaded a new version of the manuscript and addressed these points. We have expanded the description of participant recruitment and condensed the Analysis section, as suggested. We have also expanded the Discussion and included discussion of the sample demographics and how they compare to other studies, whilst acknowledging this as a potential limitation. We have also included discussion of broader implications (e.g. Brexit, change in Universal Credit) as suggested.

 Thank you for your thoughtful and constructive review.

# Peer review: Reviewer response for version 1

**DOI:** 10.21956/emeraldopenres.15274.r27697

**Published:** 2021-09-20

**Authors:** Pierre-Paul Audate

**Affiliations:** 1 Graduate School of Land Management and Regional Planning, Faculty of Planning, Architecture, Art and Design, Laval University, Quebec, QC, Canada

Overall, this is a well written manuscript that provides insights on the perceptions of food insecurity and well-being during the COVID-19. The paper makes an important contribution to the urban agriculture or more specifically home food growing literature. The authors found that home food growing initiatives are associated with lower food insecurity, which, in turn, improves well-being perceptions. These are interesting findings but, because it was a cross-sectional study I would recommend the authors to always discuss them within the study geographical context (to avoid generalization). A longitudinal study would give more possibility to generalize the findings. From my opinion: the paper is well-written with some minor modifications needed to improve the organization and clarify the study context, (1) It would be interesting to have a study area description section including maps of the participants localization. Is the study targeting a specific city or all the cities in the UK? It is not clear to me how the authors separated urban and rural participants inside the UK. They only mentioned that they excluded participants from outside the UK. (2) authors need to improve their argument on the well-being perceptions for example discuss other factors that might influence the positive point of view of the growers on their well-being. I would also like to see in the conclusions' section some implications of this study for UA policies in the UK.

Is the argument information presented in such a way that it can be understood by a non-academic audience?

Yes

Could any solutions being offered be effectively implemented in practice?

Yes

Is the work clearly and accurately presented and does it cite the current literature?

Yes

If applicable, is the statistical analysis and its interpretation appropriate?

I cannot comment. A qualified statistician is required.

Is real-world evidence provided to support any conclusions made?

Yes

Are all the source data underlying the results available to ensure full reproducibility?

Yes

Is the study design appropriate and is the work technically sound?

Yes

Are the conclusions drawn adequately supported by the results?

Yes

Does the piece present solutions to actual real world challenges?

Yes

Are sufficient details of methods and analysis provided to allow replication by others?

Yes

Reviewer Expertise:

Urban agriculture, Urban food systems, Urban greening, Urban planning, Urban heat island, sustainable development, Climate change

I confirm that I have read this submission and believe that I have an appropriate level of expertise to confirm that it is of an acceptable scientific standard.

# Response

**Published:** 2021-10-05

**Authors:** Bethan Mead

**Affiliations:** University of Liverpool, United Kingdom

Thank you for these comments and suggestions. We have uploaded an amended version of the manuscript addressing these. Specifically, we have focussed the Discussion on the UK context and expanded our discussion of well-being perceptions. Regarding maps of study areas/participant locations and separating urban and rural participants, we are unable to produce these as we did not assess precise locations of the participants other than confirming that they were based in the UK. We have acknowledged this as a limitation of the study and expanded the Limitations and Future Directions section of the manuscript in light of this. We have also expanded the Conclusion section to include a policy implication, as suggested. 

 Thank you for your helpful and insightful review.
